# Optical Sorter-Based Selection Effectively Identifies Soft Red Winter Wheat Breeding Lines With *Fhb1* and Enhances FHB Resistance in Lines With and Without *Fhb1*

**DOI:** 10.3389/fpls.2020.01318

**Published:** 2020-08-26

**Authors:** W. Jesse Carmack, Anthony Clark, Yanhong Dong, Gina Brown-Guedira, David Van Sanford

**Affiliations:** ^1^ Department of Plant and Soil Science, University of Kentucky, Lexington, KY, United States; ^2^ Department of Plant Pathology, University of Minnesota, St. Paul, MN, United States; ^3^ Plant Science Research Unit, USDA-Agricultural Research Service, Raleigh, NC, United States

**Keywords:** fusarium head blight (FHB), optical sorter, deoxynivalenol (DON), SSR markers, soft red winter wheat, fusarium damaged kernels (FDK), disease resistance, KASP markers

## Abstract

Previous results from our lab have shown that using an optical sorter to identify *Fusarium* head blight (FHB) resistant breeding lines was effective at reducing the toxin deoxynivalenol (DON) and FHB-associated kernel damage. In this paper we quantified the proportion of desirable genotypes at FHB resistance QTL in lines from three selection cycles of optical sorting. Breeding lines were genotyped at loci on chromosomes 3BS, 2DL, and 5A using the following DNA markers: TaHRC, CFD233, and GWM304. TaHRC is a KASP marker for *Fhb1*, a major FHB resistance QTL on chromosome 3BS. CFD233 is an SSR marker for Qfhs.nau-2DL on chromosome 2DL. GWM304 is an SSR marker for Qfhs.ifa-5A on chromosome 5A. Sorter selection was effective at identifying lines that had the resistant genotype at TaHRC; in other words, the sorter was able to identify lines with resistance alleles at *Fhb1*. The sorter was less effective at selecting for the resistant genotype at CFD233 and GWM304. However, the proportion of lines with resistant genotypes at GWM304 did increase with additional sorter selection, just not to the degree that was observed for the *Fhb1*-associated marker. The proportion of lines with resistant alleles at CFD233 did not show a consistent trend. In addition to increasing the proportion of lines with *Fhb1* and Qfhs.ifa-5A each selection cycle, optical sorter-based mass selection enhanced FHB resistance in different marker genotype combinations evaluated in this study. For example, there were net reductions in DON and kernel damage after two cycles of sorter selection in 15X110601S07002, a line with *Fhb1*, with Qfhs.nau-2DL, and with Qfhs.ifa-5A; final C_3_ DON levels were 63% of the resistant check (KY02C-3005-25). Kernel damage was also reduced in 15X110601A08221 a line without *Fhb1*, without Qfhs.nau-2DL, and without Qfhs.ifa-5A. Our findings suggest the increased resistance observed in different marker genotype combinations was conferred by QTL other than *Fhb1*, QFhs.nau-2DL, and Qfhs.ifa-5, and validate our previous results that the optical sorter is effective at selecting FHB-resistant breeding material.

## Introduction

Wheat (*Triticum aestivum* L.), considered a staple crop in numerous cultures, is widely consumed around the world. The Foreign Agricultural Service (FAS) a division of the United States Department of Agriculture (USDA), projects global consumption of wheat to be over 750,000,000 metric tons in the 2019/2020 marketing year (USDA FAS, 2020). As the world population and affluence continue to increase, the demand for staple crops like wheat is also expected to rise. Shiferaw et al. predicts a 60% increase in demand for wheat by the year 2050 (Shiferaw et al., 2013). Increasing wheat production to levels adequate enough to meet demand, while also mitigating contamination and other grain quality issues caused by plant diseases, is a major problem facing agricultural researchers today.

*Fusarium* head blight (FHB), caused by *Fusarium graminearum*, is a plant disease that limits wheat production and contaminates grain. Yield reduction, due to FHB-associated kernel damage, directly limits wheat production (Agostinelli et al., 2011). Damaged kernels also decrease market value due to reduced test weight and flour yield (McMullen et al., 1997). Deoxynivalenol (DON) accumulates in grain as a result of FHB infection; and, consumption of grain contaminated with DON is harmful to both humans and animals. DON toxicity symptoms include: nausea, vomiting, diarrhea, abdominal pain, headache, dizziness, fever, and, with enough exposure, death (Sobrova et al., 2010). Furthermore, no single management practice has provided complete suppression of FHB infection (Zhu et al., 2019). The most effective fungicide regimes provide at best 69% control for kernel damage and 54% control for DON (D’Angelo et al., 2014). Therefore, enhancing genetic resistance to FHB *via* plant breeding is the most promising solution to DON accumulation and kernel damage.

Quantitative trait loci (QTL) involved in FHB resistance have been identified on all 21 wheat chromosomes (Steiner et al., 2017). Two of the strongest and best-validated are *Fhb1* and Qfhs.ifa-5A, both of which were derived from “Sumai-3” (Bai et al., 1999; Waldron et al., 1999; Anderson et al., 2001; Buerstmayr et al., 2003). *Fhb1* was first described by Waldron et al. as Qfhs.ndsu-3B and later renamed (Waldron et al., 1999; Liu et al., 2006; Agostinelli et al., 2011). *Fhb1* is a major effect QTL that confers strong Type II resistance to FHB in wheat and other small grains (Bai et al., 1999; Cuthbert et al., 2006; Petersen et al., 2017; Steiner et al., 2017; He et al., 2018; Zhu et al., 2019). Qfhs.ifa-5A contributes mainly to Type I FHB resistance (Buerstmayr et al., 2003; Steiner et al., 2019). An additional large effect resistance QTL is Qfhs.nau-2DL identified in the breeding line CJ9306 (Jiang et al., 2007a; Jiang et al., 2007b; Steiner et al., 2017). Qfhs.nau-2DL contributes to both Type I and II resistance (Agostinelli et al., 2011; Lu et al., 2013; Yi et al., 2018). Type I FHB resistance is defined as resistance to initial infection, whereas Type II is defined as resistance to disease spread within infected heads (Mesterhazy, 1995; Mesterházy et al., 1999).

*Fhb1*, Qfhs.ifa-5A, and Qfhs.nau-2DL all have relatively stable effects and tightly linked DNA markers; thus, marker assisted selection (MAS) for these QTL has efficiently improved FHB resistance in adapted, high-yielding wheat germplasm (Steiner et al., 2017). In addition, wheat lines with acceptable FHB resistance can be developed through accumulation of several small effect QTL present in locally adapted germplasm, i.e. what is often termed “native resistance” (Steiner et al., 2017). Accumulating numerous small effect QTL in genetic backgrounds fixed for known major effect QTL should enhance FHB resistance in wheat germplasm and potentially other small grains. Therefore, a high-throughput selection method is needed to gradually accumulate new small effect QTL while also enriching for major effect FHB resistance QTL.

Optically separating diseased from non-diseased grain has been shown to have potential as an en masse selection method to identify and enhance FHB resistance (reduce DON and kernel damage) in wheat (Carmack et al., 2019). The objective of this study was to determine if optically sorting seed from breeding material segregating visually for FHB resistance over several generations increased the proportion of lines with resistance alleles at large effect QTL on 3BS, 2DL, and 5A. In addition to assessing the proportion of lines with the FHB resistance QTL each selection cycle, the average response to selection of all marker genotype combinations was examined, and the ability of optical sorter-based mass selection to enhance FHB resistance in individual lines with and without the R alleles at the three large effect QTL was demonstrated.

## Materials and Methods

### Plant and Fungal Material

The plant material used in this study consisted of 300 F_4_ derived soft red winter wheat (SRWW) breeding lines with KY06C-11-3-10 (Reg. No. GP-965, PI 669817) in their pedigree. KY06C-11-3-10 is a SRWW germplasm line that carries exotic FHB resistance alleles from the Chinese spring wheat cultivar “Ning7840” at QTL on chromosomes 3BS, 5A, and 2DL; the line was created *via* accelerated backcrossing of these QTL into “McCormick,” a domestic cultivar with nonexotic (native) moderate FHB resistance (Clark et al., 2014). Backcrossing was performed by the University of Kentucky, University of Maryland, Virginia Polytechnic Institute and State University, North Carolina State University, and the USDA-ARS. The breeding lines used in this study differed in characteristics such as level of FHB resistance, heading date, height, and other agronomic traits. All plant material was grown at the University of Kentucky Spindletop Research Farm near Lexington, KY (38°7'37.81''N, 84°29 44.85'' W) from 2016 to 2019.

The fungal material used in this study consisted of inoculum prepared using 27 F*. graminearum* isolates taken from scabby wheat seed collected at multiple locations across Kentucky, 2007–2010 (Bec et al., 2015). Inoculum was prepared by first allowing dry corn (*Zea mays* L.) kernels to imbibe water for approximately 16 hours. After 16 hours, corn kernels were autoclaved, inoculated with potato dextrose agar (PDA) plugs infected with *F. graminearum*, mixed with 0.2 g of streptomycin in 50 mL of sterile water, covered and allowed to incubate at room temperature (Balut et al., 2013). After complete colonization by the fungus (3 weeks), the corn kernels were spread onto a tarp and allowed to dry aided by a dehumidifier. After drying, inoculated corn kernels were placed in mesh bags and stored in a freezer at −18°C. All cultures were maintained, and inoculum was produced at the University of Kentucky Plant Science Building in Lexington, KY (38°1'36.1''N, 84°30'30.1''W) from 2016 to 2019.

### Phenotyping and Genotyping Plant Material

The breeding lines were phenotyped in an inoculated and irrigated scab nursery. The nursery provided the intense disease pressure needed for resistance evaluation and artificial selection. During all years of the experiment (2016–2019), at Feekes growth stage 8, approximately 21 days prior to flowering of the earliest material, corn kernels infected with *F. graminearum* were broadcast throughout the nursery at a rate of 11.86 g m^−2^ (Gilbert and Woods, 2006; Balut et al., 2013). In addition to inoculating the field, an overhead irrigation system on an automatic timer was used to provide optimal moisture conditions for disease development, and the opportunity to evaluate and select for FHB resistance. The irrigation schedule was as follows: 5-minute periods every 15 minutes from 2000 to 2045 h, 2100 to 2145 h, 0200 to 0245 h, 0500 to 0530 h, and 0830 h (Balut et al., 2013). The concentration of deoxynivalenol (DON) in ppm was determined by the University of Minnesota DON testing lab using gas chromatography with mass spectrometry GC-MS (Mirocha et al., 1998; Fuentes et al., 2005) each season. The proportion of *Fusarium* damaged kernels in a given sample estimated using an optical seed sorter (FDKos) was collected in all years except 2016; FDKos estimates were arrived at using methods developed by the University of Kentucky Wheat Breeding Program (Carmack et al., 2019).

The breeding lines were genotyped at the Eastern Regional Small Grains Genotyping Laboratory in Raleigh, North Carolina, USA. DNA was isolated from each of the 300 breeding lines and genotypes were determined at FHB resistance QTL using two SSR markers (Benson et al., 2012). The SSR markers used were as follows: CFD233 and GWM304. CFD233 is a SSR marker for QFhs.nau-2DL, a FHB resistance QTL on chromosome 2DL (Guyomarc’h et al., 2002; Löffler et al., 2009; Agostinelli et al., 2011; Balut et al., 2013; Kollers et al., 2013; Arruda et al., 2016). GWM304 is a SSR marker for Qfhs.ifa-5A, a FHB resistance QTL on chromosome 5A (Roder et al., 1998; Chen et al., 2006; Liu et al., 2007; Arruda et al., 2016). In addition to the SSR markers, genotypes were obtained for 120 of the 300 breeding lines using one KASP marker (TaHRC); reactions were done following the manufacturer’s instructions. TaHRC is a KASP marker for *Fhb1* on chromosome 3BS (Bernardo et al., 2011; Schweiger et al., 2016; Su et al., 2019). Previous findings from numerous labs have shown that DNA markers linked to *Fhb1* are associated with material more resistant to DON accumulation (Roder et al., 1998; Waldron et al., 1999; Anderson et al., 2001; Zhou et al., 2002; Anderson et al., 2007; Wilde et al., 2007; Liu et al., 2008; Salameh et al., 2010; Balut et al., 2013; Arruda et al., 2016; Prat et al., 2017).

### Optical Sorter-Based Mass Selection

**Figure 1:**
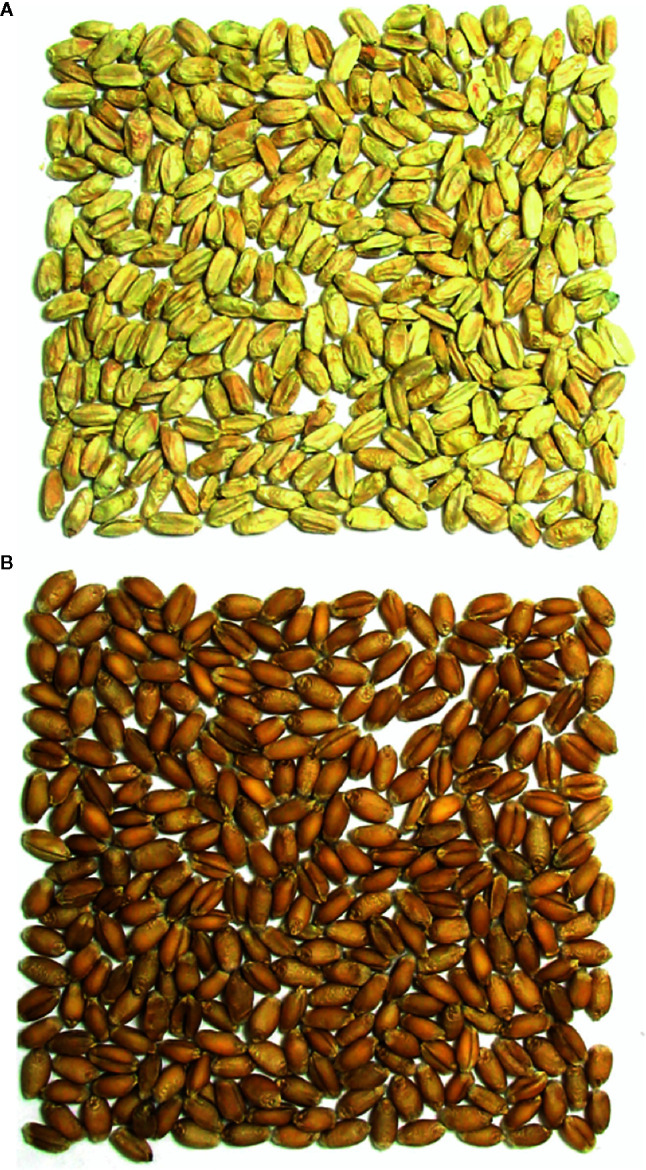
Visual differences between *Fusarium* damaged and asymptomatic kernels used to calibrate the optical sorter. **(A)** *Fusarium* damaged/rejected kernels, **(B)** asymptomatic/accepted kernels.

The optical sorter is a USDA/ARS and National Manufacturing Seed Sorter System that uses a high-throughput, high-resolution color camera in combination with compressed air to separate grain (Pasikatan and Dowell, 2003; Delwiche et al., 2005; Pearson et al., 2008; Pearson, 2010). Each cycle of mass selection with the optical sorter was performed in a different year; therefore, the sorter was calibrated each year. Optical sorter calibration and operation was performed as described in Carmack et al., 2019. **Figure 1** provides an example of the visual differences between *Fusarium* damaged (rejected) and asymptomatic (accepted) kernels used to calibrate the optical sorter.

Experimental material was grown from 2016 to 2018 at Spindletop Research Farm in 1 meter (m) rows spaced 30 centimeters (cm) apart arranged in a randomized complete block design (RCBD) with one resistant (KY02C-3005-25) and one susceptible (Pioneer Brand 2555) check cultivar repeated throughout the nursery. Cycles of selection 1, 2, and 3 (C_1_; C_2_; C_3_) were evaluated collectively in 2019 at the same location using 1 m long six row miniplots arranged in a RCBD with the resistant and susceptible checks repeated throughout the field. Three replications per genotype were evaluated in 2018 whereas two replications were used in all other years.

During mass selection (2016–2018), each 1 m row was hand harvested with sickles and all plants in each row were bundled together to avoid mixing. Each bundle was threshed separately using a stationary threshing machine, and seed from all plants in the bundle were collected in bulk and optically sorted. After sorting, grain “accepted” by the sorter from each line/rep combination was comingled, sampled and then used to plant the subsequent generation. From 2016 to 2018, all lines were advanced to the next generation after sorting (within-line selection). In 2019, cycles 1, 2, and 3 from 54 of the 300 F_4_ derived lines were evaluated in an inoculated and irrigated head scab nursery. These lines were chosen because there was enough remnant seed from previous cycles of selection (C_1_ – C_3_) to allow all 3 generations to be planted collectively in 6 row miniplots in the head scab nursery. No remnant seed from the base population (C_0_) was available, and therefore C_0_ was not grown in 2019. In addition to within-line selection, among-line selection was retroactively performed using phenotypic measurements obtained with the optical sorter (FDKos); among line selection decisions were based on FDKos values obtained from 2016–2018. For among-line selection, candidates that had FDKos values greater than the resistant check (KY02C-3005-25) were dropped each cycle of selection. No among line selection was performed from C_0_ to C_1_, because FDKos was not recorded in 2016. From C_1_ to C_2_, and C_2_ to C_3_, any lines that had FDKos values greater than the resistant check were discarded.

### Data Analysis

Mean DON and FDKos values were estimated for each breeding line by year using data collected during mass selection (2016-2018) and the following model:

$${\text{Y}}_{\text{ijk}}=\mu +{\text{Y}}_{\text{i}}+\text{R}{\left(\text{Y}\right)}_{\text{ij}}+{\text{L}}_{\text{k}}+{\text{Y}}_{\text{i}}\times {\text{L}}_{\text{k}}+\epsilon_{\text{ijk}}$$

where Y_ijk_ = the observation in the *i*th year in the *j*th rep of the *k*th breeding line, µ = the overall mean, Y_i_ = the effect of the *i*th year, R_j_ = the effect of the *j*th replication within the *i*th year, L_k_ = the effect of the *k*th breeding line, Y_i_ × L_k_ = the effect of the interaction of the *i*th year and the *k*th breeding line, ε_ijk_ = the residual error. Since each year represents a different cycle of selection, it was necessary to nest replications in years. The model used to determine mean DON and FDKos values for each breeding line, marker genotype, and selection cycle using data collected during the final evaluation in 2019 was:

$${\text{Y}}_{\text{ijk}}=\mu +{\text{C}}_{\text{i}}+{\text{R}}_{\text{j}}+{\text{G}}_{\text{k}}+{\text{C}}_{\text{i}}\times {\text{G}}_{\text{k}}+\epsilon_{\text{ijk}}$$

where Y_ijk_ = the observation in the *i*th cycle in the *j*th rep of the *k*th breeding line or marker genotype, µ = the overall mean, C_i_ = the effect of the *i*th selection cycle, R_j_ = the effect of the *j*th replication, G_k_ = the effect of the *k*th breeding line or marker genotype, C_i_ × G_k_ = the effect of the interaction of the *i*th selection cycle and the *k*th breeding line or marker genotype, ε_ijk_ = the residual error.

## Results

### Optical Sorter-Based Among Line Selection Increased the Proportion of Breeding Lines With *Fhb1*

The results of FDKos among line selection presented in **Table 1** are based on data obtained during the final evaluation (2019). Among line selection with the optical sorter resulted in a net decrease in overall DON concentration each cycle; overall FDKos values also decreased with each cycle of selection (Carmack et al., 2019). In addition to the observed decrease in cycle mean DON and FDKos, the proportion of TaHRC resistant (R) genotypes increased with each additional round of sorter-based among line selection. Specifically, the proportion of lines with *Fhb1* went from 48% to 92% after two rounds of sorter-based among line selection. Average cycle DON and FDKos for TaHRC R genotypes ranged from 0.7 to 0.8 ppm and 12.8 to 19.2% respectively. Not only did the proportion of TaHRC R genotypes increase, the proportion of TaHRC susceptible (S) genotypes decreased with additional cycles of optical sorter-based among line selection. When the study was initiated, prior to optical sorting, 37% of lines did not have *Fhb1*, and after two cycles of sorter-based among line selection 100% of the lines had at least one R allele at *Fhb1.* Average DON concentration was always higher in TaHRC S genotypes than in TaHRC heterozygous (H) or R genotypes and ranged from 1.6 to 1.8 ppm. FDKos ranged from 17.5 to 23.5%. The proportion of TaHRC H genotypes decreased each round of sorter-based among line selection (from 15 to 12 to 8%). Mean DON concentrations for TaHRC H genotypes ranged from 0.3 to 0.8 ppm and FDKos values ranged from 19.7 to 25.1%.

**Table 1:** Shift in genotype proportions by DNA marker and selection cycle due to optical sorter-based (FDKos) mass selection estimated using data collected during the final evaluation (2019), Lexington, KY.

					TaHRC (3BS)			CFD233 (2DL)			GWM304 (5A)		
C	N	Cycle FDKos	Cycle DON	G	P	FDKos	DON	P	FDKos	DON	P	FDKos	DON
C_1_	54	20.9 ± 1.2	1.1 ± 0.1	R	48	19.2 ± 1.9	0.8 ± 0.1	35	21.6 ± 2.2	1.1 ± 0.2	43	21.1 ± 2.0	0.9 ± 0.2
				H	15	19.7 ± 3.4	0.8 ± 0.3	9	20.2 ± 4.4	1.5 ± 0.4	11	23.7 ± 4.0	1.2 ± 0.3
				S	37	23.5 ± 2.1	1.6 ± 0.2	56	20.5 ± 1.8	1.0 ± 0.2	46	20.0 ± 1.9	1.3 ± 0.2
C_2_	25	18.1 ± 1.8	0.9 ± 0.2	R	72	17.1 ± 1.9	0.7 ± 0.1	32	18.0 ± 2.9	0.7 ± 0.2	44	20.7 ± 2.4	0.7 ± 0.2
				H	12	25.1 ± 4.5	0.8 ± 0.3	8	14.9 ± 5.9	2.2 ± 0.4	12	14.4 ± 4.6	1.1 ± 0.4
				S	16	17.5 ± 3.9	1.8 ± 0.3	60	18.6 ± 2.1	0.8 ± 0.2	44	16.5 ± 2.4	0.9 ± 0.2
C_3_	12	13.7 ± 2.6	0.7 ± 0.2	R	92	12.8 ± 1.8	0.7 ± 0.2	33	9.0 ± 2.9	0.5 ± 0.4	50	17.5 ± 2.3	0.9 ± 0.3
				H	8	23.6 ± 5.9	0.3 ± 0.7	8	10.8 ± 5.8	1.1 ± 0.7	8	10.8 ± 5.6	0.2 ± 0.7
				S	0	N/A	N/A	59	16.7 ± 2.2	0.7 ± 0.3	42	9.6 ± 2.5	0.5 ± 0.3

TaHRC, KASP marker for Fhb1 on chromosome 3BS; CFD233, SSR marker for FHB resistance QTL on chromosome 2DL; GWM304, SSR marker for FHB resistance QTL on chromosome 5A; C, selection cycle, C_1_, 1^st^ cycle of selection; C_2_, 2nd cycle of selection; C_3_, 3rd cycle of selection; N, number of lines selected in previous generation; G, genotype; R, resistant genotype; H, heterozygous genotype; S, susceptible genotype, P, the percentage of breeding lines each cycle with the corresponding genotype; ±, standard error of the mean; FDKos, Fusarium damaged kernels determined using an optical sorter (%); DON, deoxynivalenol (ppm).

### Optical Sorter-Based Among Line Selection Increased the Proportion of Breeding Lines With FHB Resistance QTL on Chromosome 5A and Not 2DL

Similar to the pattern observed for TaHRC, optical sorter-based among line selection resulted in a net increase in the proportion of lines with R genotypes at GWM304 (the SSR marker for Qfhs.ifa-5A) each cycle of sorter selection (**Table 1**). The proportion of lines with GWM304 R genotypes went from 43 (C_1_) to 50% (C_3_). Mean DON for GWM304 R genotypes ranged from 0.7 to 0.9 ppm, and mean FDKos ranged from 17.5 to 21.1%. Furthermore, the proportion of lines with S genotypes for GWM304 decreased each cycle of selection: C_1_ = 46%, C_2_ = 44%, and C_3_ = 42%. In other words, sorter-based among line selection resulted in a shift from mostly S genotypes in C_1_ to majority R genotypes in C_3_. This is a promising result. For GWM304 S genotypes, mean DON and FDKos ranged from 0.5 to 1.3 ppm and 9.6 to 20.0% respectively. The proportion of GWM304 H genotypes increased (11 to 12%) after one round of sorter-based among line selection and then decreased (12 to 8%) with an additional round of selection. Mean DON concentrations for GWM304 H genotypes ranged from 0.2 to 1.2 ppm and FDKos values ranged from 10.8 to 23.7%.

In contrast with TaHRC (the KASP marker for *Fhb1*) and GWM304 (the SSR marker for Qfhs.ifa-5A), the proportion of lines with CFD233 R genotypes did not consistently increase and S genotypes did not decrease with additional optical sorter-based among line selection (**Table 1**). The proportion of R, H, and S genotypes for the QFhs.nau-2DL marker (CFD233) remained fairly constant with additional optical sorter based-among line selection. Specifically, CFD233 R genotypes went from 35 to 33%, S genotypes went from 56 to 59%, and H genotypes went from 9 to 8% after two rounds of sorter-based among line selection. DON ranged from 0.5 to 1.1 ppm for R, 0.7 to 1.0 ppm for S, and 1.1 to 2.2 ppm for H genotypes. FDKos ranged from 9.0 to 21.6% for R, 16.7 to 20.5% for S, and 10.8 to 20.2% for H genotypes.

### Phenotypic Variation Among Different Marker Genotype Combinations Indicate Optical Sorter-Based Within Line Mass Selection Enhanced FHB Resistance in Some Genetic Backgrounds

The results of optical sorter-based within line mass selection presented in **Tables 2** and **3** are based on data obtained during the final evaluation in 2019. Contrary to what was observed for optical sorter-based among line selection, sorter-based within line selection did not result in a net decrease in overall DON and FDKos with each additional cycle of selection (Carmack et al., 2019). However, specific marker genotype combinations did respond to within line selection (**Table 2**). Of the 54 lines tested in 2019, 19 were heterozygous for at least one marker. The other 35 lines fit into one of eight possible genotypes (RRR, RRS, RSS, RSR, SSS, SRR, SSR, and SRS), where the first letter represents the genotype at TaHRC, the second represents CFD233, and the third represents GWM304. Net reductions in FHB-associated kernel damage (FDKos) were achieved in RRR, RRS, RSS, SSS, SSR, and SRS genotypes. Mean FDKos ranged from 7.1 to 32.2%. Similar to what was observed with FDKos, sorter-based within line selection resulted in lower net DON values in RRS, SSR, and SRS genotypes. Mean DON ranged from 0.5 to 2.2 ppm.

**Table 2:** Means and standard errors for DON and FDKos for all marker genotype combinations by selection cycle estimated using phenotypic data collected during the final evaluation (2019), Lexington, KY.

Cycle	3BS	2DL	5A	FDKos	FDKos as % KY02C-3005-25	DON	DON as % KY02C-3005-25
C_1_	R	R	R	31.2 ± 2.5	328	1.0 ± 0.4	128
C_2_	R	R	R	32.2 ± 2.5	339	0.9 ± 0.4	124
C_3_	R	R	R	27.0 ± 2.5	284	1.0 ± 0.4	128
C_1_	R	R	S	14.6 ± 3.9	154	1.1 ± 0.2	141
C_2_	R	R	S	16.5 ± 3.9	173	0.8 ± 0.2	106
C_3_	R	R	S	13.4 ± 3.9	141	0.6 ± 0.2	84
C_1_	R	S	S	17.1 ± 5.6	180	0.5 ± 0.2	65
C_2_	R	S	S	20.4 ± 5.6	215	0.5 ± 0.2	65
C_3_	R	S	S	13.8 ± 5.6	145	0.5 ± 0.2	65
C_1_	R	S	R	18.0 ± 3.6	189	0.6 ± 0.4	82
C_2_	R	S	R	17.2 ± 3.6	181	0.8 ± 0.4	102
C_3_	R	S	R	18.6 ± 3.6	196	0.8 ± 0.4	102
C_1_	S	S	S	22.3 ± 2.4	235	1.3 ± 0.3	171
C_2_	S	S	S	22.5 ± 2.4	236	1.5 ± 0.3	197
C_3_	S	S	S	18.1 ± 2.4	191	1.4 ± 0.3	180
C_1_	S	R	R	7.1 ± 2.5	74	0.5 ± 0.2	64
C_2_	S	R	R	10.8 ± 2.5	114	0.7 ± 0.2	85
C_3_	S	R	R	13.6 ± 2.5	143	0.6 ± 0.2	81
C_1_	S	S	R	22.8 ± 3.8	240	1.5 ± 0.3	193
C_2_	S	S	R	18.8 ± 3.8	198	1.5 ± 0.3	193
C_3_	S	S	R	20.5 ± 3.8	215	1.2 ± 0.3	155
C_1_	S	R	S	27.4 ± 7.3	288	2.2 ± 0.7	283
C_2_	S	R	S	25.8 ± 7.3	271	1.9 ± 0.7	246
C_3_	S	R	S	26.7 ± 7.3	282	2.1 ± 0.7	276

Cycle, selection cycle; C_1_, 1st cycle of selection; C_2_, 2nd cycle of selection; C_3_, 3rd cycle of selection; 3BS, FHB resistance QTL on chromosome 3BS (Fhb1); 2DL, FHB resistance QTL on chromosome 2DL (QFhs.nau-2DL); 5A, FHB resistance QTL on chromosome 5A (Qfhs.ifa-5A); R, resistant genotype; S, susceptible genotype; ±, standard error of the mean; FDKos, Fusarium damaged kernels determined using an optical sorter (%); DON, deoxynivalenol (ppm); KY02C-3005-25, resistant check cultivar.

**Table 3:** Response of individual lines to optical sorter-based within line selection by selection cycle estimated using phenotypic data collected during the final evaluation (2019), Lexington, KY.

Line	Genotype(3BS, 2DL, 5A)	Cycle	FDKos	FDKos as %KY02C-3005-25	DON	DON as %KY02C-3005-25
15X110601S07002	RRR	C_1_	31.6 ± 4.9	332	0.7 ± 0.2	86
		C_2_	28.8 ± 4.9	303	0.7 ± 0.2	86
		C_3_	21.3 ± 4.9	224	0.5 ± 0.2	63
15X110601A08053	RRS	C_1_	37.5 ± 2.3	394	1.6 ± 0.5	208
		C_2_	37.1 ± 2.3	390	1.0 ± 0.5	127
		C_3_	36.5 ± 2.3	384	0.7 ± 0.5	93
15X110599S05047	RSS	C_1_	11.9 ± 1.0	125	0.3 ± 0.0	41
		C_2_	15.2 ± 1.0	159	0.3 ± 0.0	41
		C_3_	8.9 ± 1.0	93	0.2 ± 0.0	24
15X110599A06069	RSR	C_1_	22.9 ± 1.5	241	1.0 ± 0.3	126
		C_2_	13.5 ± 1.5	142	0.9 ± 0.3	116
		C_3_	20.5 ± 1.5	216	0.5 ± 0.3	63
15X110601A08221	SSS	C_1_	36.9 ± 2.2	388	1.1 ± 0.5	145
		C_2_	25.6 ± 2.2	269	2.3 ± 0.5	303
		C_3_	18.3 ± 2.2	192	2.1 ± 0.5	270
15X110599S05176	SRR	C_1_	7.1 ± 2.5	74	0.5 ± 0.2	64
		C_2_	10.8 ± 2.5	114	0.7 ± 0.2	86
		C_3_	13.6 ± 2.5	143	0.6 ± 0.2	81
15X110601S07085	SSR	C_1_	13.7 ± 0.3	144	1.6 ± 0.3	204
		C_2_	9.2 ± 0.3	97	1.2 ± 0.3	160
		C_3_	12.9 ± 0.3	135	0.8 ± 0.3	105
15X110601A08142	SRS	C_1_	41.1 ± 3.1	432	3.7 ± 0.4	480
		C_2_	27.6 ± 3.1	291	1.5 ± 0.4	197
		C_3_	35.8 ± 3.1	376	2.5 ± 0.4	322

Line, experimental name for a specific F_4_ derived University of Kentucky SRWW breeding line; genotype, the genotype combination at the marker loci on 3BS, 2DL, and 5A; R, resistant genotype; S, susceptible genotype; cycle, selection cycle; C_1_, 1st cycle of selection; C_2_, 2nd cycle of selection; C_3_, 3rd cycle of selection; ±, standard error of the mean; FDKos, Fusarium damaged kernels determined using an optical sorter (%); DON, deoxynivalenol (ppm); KY02C-3005-25, resistant check cultivar.

### At Least One Individual Line in Each of the Eight Marker Genotype Combinations Responded to Optical Sorter-Based Within Line Mass Selection

Individual lines in each of the eight marker genotype combinations responded to within line selection with the optical sorter. Mean DON and FDKos by cycle of selection for one individual line that responded to optical sorter-based within line selection from each marker genotype combination is presented in **Table 3**. Unexpectedly, DON and FDKos values increased each cycle of selection in line 15X110599S05176, an SRR genotype. Line 15X110599S05176 was the only SRR genotype evaluated in 2019; it is possible that a different SRR line may have responded positively to selection (DON and FDKos lowered with additional selection). For the other seven genotype combinations (RRR, RRS, RSS, RSR, SSS, SSR, and SRS) the individual line represented in **Table 3** shows a net decrease in at least one target trait (DON and/or FDKos) each additional cycle of within line sorter selection. FDKos values ranged from 7.1 to 41.1% and DON concentrations from 0.2 to 3.7 ppm.

## Discussion

On the basis of our previously published results and the results of this study, it is our opinion that optical seed sorter-based selection has potential to enhance FHB resistance (reduce DON accumulation and kernel damage) in SRWW. In this paper, we determined the effectiveness of the sorter at identifying breeding material with known FHB resistance QTL on chromosomes 3BS, 2DL, and 5A, by evaluating the proportion of desirable genotypes at the QTL in lines from three selection cycles of optical sorting. In addition to assessing the proportion of lines with the FHB resistance QTL on 3BS, 2DL, and 5A each selection cycle, the average response to selection of all marker genotype combinations was examined. Furthermore, the ability of optical sorter-based mass selection to enhance FHB resistance in individual lines with and without the R alleles at the FHB resistance QTL was demonstrated.

Sorter selection was very effective at identifying lines that had the resistant genotype at TaHRC; in other words, the sorter was able to identify lines with resistance alleles at *Fhb1* (**Table 1**). In addition to a net decrease in overall DON and FDKos each cycle, we observed that sorter-based among line selection resulted in an increased proportion of TaHRC R genotypes and a decreased proportion of S genotypes with each additional round of selection. These results are not unexpected. The majority of major effect QTL detected for reduced DON accumulation, including *Fhb1*, co-located with QTL for reduced disease severity on plants preharvest or grains postharvest (Somers et al., 2003; Liu et al., 2012; Liu et al., 2013; Ágnes et al., 2014; Buerstmayr and Lemmens, 2015). Furthermore, *Fhb1* has been classified as a strong contributor to Type II FHB resistance, which is associated with reductions in *Fusarium* damaged kernels (Bai et al., 1999; He et al., 2018). The proportion of *Fusarium* damaged kernels determined with an optical sorter (FDKos) is a postharvest measure of disease severity on grain as well as an indicator of Type II FHB resistance; therefore, it is not surprising that optical sorter-based among line selection (lines with FDKos values greater than the resistant check were discarded each selection cycle) increased the proportion of lines with *Fhb1* (a QTL known to be associated with reduced kernel damage). The increased proportion of lines with *Fhb1*, a QTL that has been shown time after to time to enhance head scab resistance in wheat and other small grains, validates our previous findings that optical sorter-based among line selection can be utilized to breed for lower DON and FHB-associated kernel damage (Carmack et al., 2019).

The proportion of lines with a resistant genotype at GWM304 increased with additional sorter selection to a lesser degree than what was observed for TaHRC (**Table 1**). We expected sorter-based among line selection to increase the proportion of GWM304 R genotypes similar to the pattern we saw with TaHRC, considering that Qfhs.ifa-5A, like *Fhb1*, is classified as a large effect FHB resistance QTL associated with reduced disease severity and DON content (Buerstmayr and Lemmens, 2015). Previous studies have proposed a role for Qfhs.ifa-5A in reduced disease severity on plants preharvest or grains postharvest; however, different methods for estimating disease severity were used (Miedaner et al., 2006; Wilde et al., 2007; Kang et al., 2011). Miedaner et al., and Wilde et al. estimated FHB disease severity using a rating scale on plants preharvest, whereas Kang et al. estimated disease severity using a visual estimate of the percentage of *Fusarium* damaged kernels in a sample of grain postharvest (FDK). Rating was a visual estimate of the proportion of diseased heads in a plot from 0 to 9, where 0 = no heads showing disease symptoms and 9 = 90% of heads showing disease symptoms. FDK was estimated as the percentage of visually infected kernels in a sample, which included shriveled and discolored seeds. Both methods were different than disease severity determined with an optical sorter (FDKos). Optical sorter-based selection operated on detectable differences in seed color only, not seed shape/size or preharvest appearance of the plant. Furthermore, previous research has shown the QTL on 5A contributes mostly to Type I resistance, unlike *Fhb1* which contributes to strong Type II resistance (Bai et al., 1999; Buerstmayr et al., 2003; He et al., 2018; Steiner et al., 2019). Therefore, Qfhs.ifa-5A may be more involved in reducing physical kernel damage (shriveled seeds/FDK) and visible preharvest disease symptoms (rating), than differences in seed coat color as a result of FHB infection (FDKos). It appears *Fhb1* may be heavily involved in expression of all three traits. This would explain the inability of the sorter to select for GWM304 R genotypes to the same degree as for TaHRC R genotypes. Although sorter selection did not increase the proportion of lines with R genotypes at GWM304 as was observed with TaHRC, sorter-based among line selection resulted in a shift from mostly S genotypes in C_1_ to majority R genotypes in C_3_. The results presented in **Table 1** for GWM304 agree with those for TaHRC and support the notion that optical sorter-based among line selection can be used to select for R genotypes and against S genotypes at known FHB resistance QTL.

The sorter was not effective at selecting for the resistant genotype at the CFD233 marker locus (**Table 1**). This was unexpected, because QFhs.nau-2DL is a large effect QTL associated with reduced kernel damage and DON accumulation just like Qfhs.ifa-5A and *Fhb1* (Agostinelli et al., 2011; Balut et al., 2013). Agostinelli et al. and Balut et al. both proposed the QTL on 2DL reduced kernel damage and DON accumulation, and both estimated kernel damage using a vacuum seed sorter that separates healthy from diseased kernels on the basis of weight. Heavy kernels were considered healthy and lighter kernels were considered diseased. This method of estimating the proportion of *Fusarium* damaged kernels did not incorporate differences in seed color, which may explain the inability of sorter-based among line selection to gradually increase the proportion of R genotypes at CFD233. Interestingly, the QTL on 2DL contributes to Type I and II resistance (Agostinelli et al., 2011; Lu et al., 2013; Yi et al., 2018). Remember that the sorter effectively selected for strong Type II (*Fhb1)* and to a lesser degree Type I (QTL on 5A) resistance. Failure to select for a QTL shown to be involved in both Type I and II resistance may indicate QFhs.nau-2DL has less of an effect on FHB resistance in the genetic backgrounds utilized in this study compared to those of previously published results. Regardless, the optical sorter was not effective at selecting for lines with QFhs.nau-2DL (the proportion of lines with CFD233 R genotypes did not increase with additional rounds of selection) in our selection material.

Although optical sorter-based among line selection did not result in genotype proportions equal to optimum MAS results for all three markers, 92% of C_3_ lines selected based on FDKos had *Fhb1*, compared to 48% in the C_1_. These proportions are comparable to the best MAS results, in which 100% of lines would have the marker. Sorter-based among line selection resulted in a shift from mostly S genotypes in C_1_ (R = 43%; S = 46%) to majority R genotypes in C_3_ (R = 50%; S = 42%) for the QTL on 5A; unfortunately, the proportion of R and S genotypes for the QTL on 2DL did not show a consistent response. MAS would have resulted in a shift to all R genotypes. Therefore, MAS outperformed sorter-based among line selection at increasing the proportion of R genotypes at the three FHB resistance QTL. However, it did not reduce DON and FDKos values lower than sorter-based among line selection. For example, MAS (selecting only RRR lines) resulted in final DON concentrations greater than and final FDKos values significantly greater than those of sorter-based among line selection: DON = 1.0 ± 0.4 ppm and FDKos = 27.0 ± 2.5% (**Table 2**) compared to DON = 0.7 ± 0.2 ppm and FDKos = 13.7 ± 2.6% (**Table 1**). In other words, phenotypic selection with the sorter outperformed genotypic selection with DNA markers. Phenotypic selection followed by genotypic selection did not consistently outperform phenotypic selection alone. None of the 12 C_3_ lines phenotypically selected with optical sorter-based among line selection were RRR; four lines were RRS and four lines were RSR (**Table 4**). Final mean DON and FDKos values for the RRS lines (identified using phenotypic followed by genotypic selection) were arithmetically less than those obtained *via* phenotypic selection alone (all 12 lines): DON = 0.5 ± 0.3 ppm and FDKos = 9.0 ± 1.8% (**Table 4**) compared to DON = 0.7 ± 0.2 ppm and FDKos = 13.7 ± 2.6% (**Table 1**). However, final mean DON and FDKos for the RSR lines (identified using phenotypic followed by genotypic selection) were arithmetically greater than those obtained *via* phenotypic selection alone (all 12 lines): DON = 0.9 ± 0.3 ppm and FDKos = 17.7 ± 1.8% (**Table 4**) compared to DON = 0.7 ± 0.2 ppm and FDKos = 13.7 ± 2.6% (**Table 1**). Even though optical sorter-based among line selection did not achieve genotype proportions equal to that of MAS, sorter-based selection was necessary to achieve the greatest reductions in DON and FDKos. These results support the efficacy of the optical sorter as a useful breeding tool for head scab resistance in SRWW and add merit to the idea of using an optical sorter to mass select for quantitative seed color traits in wheat and other crops (Chiou et al., 1994; Lee et al., 1998; Delwiche et al., 2005; Goggi et al., 2006; Yang et al., 2009; Pearson, 2010; Brabec et al., 2017).

**Table 4:** Average DON and FDKos by marker genotype combination for the 12 C_3_ lines phenotypically selected with the optical sorter.

Line	Genotype (3B, 2D, 5A)	FDKos	FDKos as % KY02C-3005-25	DON	DON as % KY02C-3005-25
15X110599S05115	HSR	23.6 ± 3.6	248	0.3 ± 0.5	34
15X110601S07109	RHR	10.9 ± 3.6	114	1.0 ± 0.5	145
15X110599S05057					
15X110599S05034	RRS	9.0 ± 1.8	95	0.5 ± 0.3	69
15X110599S05109					
15X110601S07003					
15X110599S05084	RSH	10.8 ± 3.6	114	0.2 ± 0.5	26
15X110599A06211					
15X110597S01102	RSR	17.7 ± 1.8	186	0.9 ± 0.3	121
15X110599A06069					
15X110599S05131					
15X110599S05036	RSS	12.0 ± 3.6	126	0.5 ± 0.5	70

Line, experimental name for a specific F_4_ derived University of Kentucky SRWW breeding line; genotype, the genotype combination at the marker loci on 3BS, 2DL, and 5A; R, resistant genotype; S, susceptible genotype; H, heterozygous genotype; ±, standard error of the mean; FDKos, Fusarium damaged kernels determined using an optical sorter (%); DON, deoxynivalenol (ppm); KY02C-3005-25, resistant check cultivar.

In addition to the observed increase in the proportion of lines with *Fhb1* and Qfhs.ifa-5A as a result of each additional optical sorter-based among line selection cycle, optical sorter-based within line selection enhanced FHB resistance in certain genetic backgrounds (**Table 2**). For example, C_1_ FDKos values went from 235% of the resistant check to 236% and 191% in the C_2_ and C_3_ generations, respectively, for lines with the SSS genotype (lines without *Fhb1* and the resistance QTL on chromosomes 2DL and 5A). These results indicate that sorter-based within line selection led to the accumulation of kernel damage resistance conferred by QTL other than the three reported in this paper, i.e. what is often termed “native resistance.” SSR genotypes (lacked *Fhb1* and 2DL) also responded to within line selection: DON accumulation was reduced from 193% of the resistant check in the C_2_ to 155% in the C_3_ (no change occurred from C_1_ to C_2_). These results agree with FDKos results for SSS lines and suggest that sorter-based within line selection accumulated head scab resistance conferred by QTL other than those evaluated in this study. In addition, breeding lines with the RRS genotype (with *Fhb1* and QFhs.nau-2DL; without Qfhs.ifa-5A) responded even more to within line selection; C_1_ DON levels went from 141% of the resistant check to 106% and 84% of the resistant check in the C_2_ and C_3_ generations (**Table 2**). C_3_ DON levels at 84% of the resistant check indicate that within line selection improved RRS genotypes on average from worse than the resistant check to better than the resistant check after just two cycles of selection. These results indicate that it is possible to use the sorter to select for resistance conferred by unknown QTL.

Furthermore, there are some very promising individual lines shown in **Table 3**. For example, line 15X110601S07002 (RRR genotype) started with C_1_ DON at 86% of the resistant check and was at 63% in C_3_. FDKos also decreased from 332 to 224% of the resistant check after two cycles of sorter selection. Response to within line selection to the degree observed for line 15X110601S07002 indicates that sorter-based within line selection led to the accumulation of FHB-associated kernel damage resistance conferred by QTL other than *Fhb1*, QFhs.nau-2DL, and Qfhs.ifa-5A. Another line, 15X110599S05047 (RSS genotype), ended up with C_3_ DON at 24% and FDKos at 93% of the resistant check. In addition, lines 15X110601A08053 (RRS genotype) and 15X110599A06069 (RSR genotype) started with C_1_ DON levels above that of the resistant check and after two rounds of selection had C_3_ DON levels below that of the resistant check. These results indicate that although optical sorter-based within line selection did not improve all traits in all lines each cycle, within line progress was accomplished. This observation, when coupled with the generation (F_4:5_) in which selection started, suggests that it may be possible to use the sorter to significantly enhance head scab resistance in wheat. Genetic variation, necessary for progress in plant breeding, is less within an F_4_ derived breeding line than, for example, an F_2_ population (Falconer, 1989). At a minimum, our results indicate further research that utilizes the optical seed sorter to enhance FHB resistance by accumulating numerous small-effect QTL (“native resistance”) is both warranted and necessary.

## Conclusion

Previous results from our lab have shown that using an optical sorter to identify FHB resistant breeding lines was effective at reducing the toxin deoxynivalenol and FHB-associated kernel damage. In this study we examined whether optical sorter-based selection increased the proportion of lines with known FHB resistance QTL, and evaluated the response to selection of the different possible marker genotype combinations, producing a few key findings:

1. Optical sorter-based among line selection increased the proportion of breeding lines with *Fhb1* and Qfhs.ifa-5A, but not QFhs.nau-2DL.
2. Phenotypic selection with the optical sorter for reduced DON and FDKos outperformed marker assisted selection (MAS); i.e., sorter-based selection was necessary to achieve the greatest reductions in DON and FDKos.
3. Optical sorter-based within line mass selection enhanced FHB resistance in certain genetic backgrounds (RRR, RRS, RSS, RSR, SSS, SRR, SSR, and SRS), which suggests the increased resistance was conferred by QTL other than *Fhb1*, QFhs.nau-2DL, and Qfhs.ifa-5A.

## Data Availability Statement

The raw data supporting the conclusions of this article will be made available by the authors, without undue reservation.

## Author Contributions

WJC helped design the study, carried out the research, analyzed data, wrote the manuscript. AC helped design the study and develop experimental materials. YD carried out mycotoxin analyses. GBG facilitated genotyping and provided genotyping data. DVS helped design the study, assisted in data analysis, edited and revised manuscript. All authors contributed to the article and approved the submitted version.

## Funding

This work was funded by a grant from the U.S. Department of Agriculture, through the US Wheat and Barley Scab Initiative under agreement no. 59-0206-9-054. The funding sponsors had no role in the design of the study; in the collection, analysis or interpretation of data; in the writing of the manuscript, and in the decision to publish the results.

## Conflict of Interest

The authors declare that the research was conducted in the absence of any commercial or financial relationships that could be construed as a potential conflict of interest.
